# Supplementary material for: Mercury deposition and redox transformation processes in peatland constrained by mercury stable isotopes
Source: Nat Commun. 2023 Nov 15;14:7389. doi: 10.1038/s41467-023-43164-8 (PMC10652010; doi:10.1038/s41467-023-43164-8)
Supplement: Supplementary file 1 — Supplementary Information [file 41467_2023_43164_MOESM1_ESM.pdf]

Supplementary text, figures, and tables

Mercury deposition and redox transformation processes in peatland constrained by mercury stable isotopes

Chuxian Li[1]*, Martin Jiskra[2], Mats B. Nilsson[1], Stefan Osterwalder[3], Wei Zhu[1], Dmitri Mauquoy[4], Ulf Skyllberg[1], Maxime Enrico[5], Haijun Peng[1], Yu Song[1], Erik Björn[6], Kevin Bishop[7]

[1]Department of Forest Ecology and Management, Swedish University of Agricultural Sciences, 90183 Umeå, Sweden
[2]Environmental Geosciences, University of Basel, 4056 Basel, Switzerland
[3]Institute of Agricultural Sciences, ETH Zurich, 8092 Zurich, Switzerland
[4]School Geosciences, University of Aberdeen, AB24 3UF Scotland, UK
[5]Universite de Pau et des Pays de l'Adour, E2S UPPA, CNRS, TotalEnergies, LFCR, IPREM, 64000 Pau, France
[6]Department of Chemistry, Umeå University, SE-90187 Umeå, Sweden
[7]Department of Aquatic Sciences and Assessment, Swedish University of Agricultural Sciences, 75007 Uppsala, Sweden
*Corresponding author (chuxian.li@slu.se)

Contents:

Supplementary Text p.2
Supplementary Figures p.4
Supplementary Tables p.11
Supplementary References p.24

Text S1. Interpretation on peat accumulation rate and Hg concentration

Hummock peat accumulation rates (AR) based on 27 radiocarbon dates were calculated to 0.035 ± 0.012 g cm^{-2} yr^{-1} in the acrotelm (above the average ground water table level of 32 ± 5 cm) and 0.010 ± 0.015 g cm^{-2} yr^{-1} in the catotelm (below the acrotelm, supplementary Table S1; Table S2; Figure S2). A higher peat AR in the acrotelm compared to the catotelm is due to the fact that the peat above the water table level has not undergone as much decomposition [1]. Compared to the hummock profile, the lawn peat AR (based on 32 radiocarbon dates) was significantly lower in the acrotelm (0.026 ± 0.004 g cm^{-2} yr^{-1} , P = 0.014, two-tailed T test, above 12 ± 5 cm) [2]. The peat AR in the lawn acrotelm is in good agreement with previous Degerö records based on validated ^{210}Pb age (0.026 ± 0.002 g cm^{-2} yr^{-1}) [3]. Both hummock and lawn records show broadly similar Hg concentration profiles with pronounced peaks between 50 cm and 30 cm depth (108 and 98 ng g^{-1}, respectively, Figure 2a, 2b). The date of the peaks, however, differ between the hummock (1960s CE) and lawn (1900s CE). In addition to atmospheric Hg deposition, peat Hg concentration is strongly influenced by decomposition. An older Hg concentration peak in the Degerö lawn than in the hummock may be due to a higher decomposition level associated with a greater mass loss [4], enabling more Hg sequestered at 1900s in lawn. This is partially supported by a lower net peat AR (0.008 ± 0.001 g cm^{-2} yr^{-1}, n = 3) compared to the hummock (0.035 ± 0.006 g cm^{-2} yr^{-1}, n = 3).

Text S2: **Hg diffusive flux between ambient air and peatland sub-surface**

We estimate the potential vertical diffusive Hg^0 flux following Fick's first law (Fs, ng m^{-3} d^{-1}, equation 1), by making use of 1/ the concentration and depth gradients between the air and peat soil gas Hg^0 concentration, and 2/ peat soil porosity and water content in our study site. Note that no advection flux is included in the calculation.

$$Fs = -Ds * \frac{\Delta C}{\Delta Z} \qquad (1)$$

where the symbol of "-" represents the efflux in the reverse direction of the concentration gradient. Ds is gas diffusion coefficient in peat soil (m^2 s^{-1}) and ΔC/ΔZ is the concentration gradient (0.45 vs 1.31 ng m^{-3}) between soil depth Z and the ambient air (−10 vs +25cm). Ds is further calculated by equation 2.

$$Ds = \xi * Da \qquad (2)$$

where ξ is the relative gas diffusion coefficient or the gas tortuosity factor. Following the approach suggested by Millington and Quick, (1961) [5], Degerö peat soil ξ is derived from equation 3.

$$\xi = (\phi - \Theta)^{\frac{10}{3}} / \phi^2 \qquad (3)$$

where ϴ is the volumetric soil water content (0.923 at a depth of ca. −7 cm below the lawn surface, Nijp et al., 2017) and ϕ is the porosity (ϕ = 100.38 − 76.7x, "x" is the average bulk density of 0.046 g cm^{-3} in the lawn acrotelm) [6].

Da is the Hg^0 diffusion coefficient in free air (m^2 s^{-1}, equation 4).

$$Da = Da_0 * \left(\frac{T}{T_0}\right)^{1.75} * \left(\frac{P}{P_0}\right) \qquad (4)$$

where T is the temperature (K), P is the air pressure in Degerö peat (0.979 × 10^5 Pa) [7] and Da^0 is a reference value of Da at T_0 (20 ◦C or 293.15 K) and P_0 (1.013 × 10^5 Pa), given as 1.47 × 10^{-5} m^2 s^{-1} in Jone, (1992) [8].

Text S3: **Peat water DGM saturation level**

Peat water DGM saturation level (S) [9] was calculated as follows.

$$S = \mathrm{Cw} * Kh/Ca \qquad (5)$$

Where Cw represents peat water DGM concentration (77 pg L^{-1}) over the period of June to July 2021 and Kh is the Henry's constant law (Kh = exp(2403.3/T + 6.92)) [10]. T is 289.5 K (16.35 °C) based on the surface ground water at Degerö from June to July 2021. Ca stands for air Hg^0 concentration (1.31 ng m^{-3}).

Text S4: Peat Hg loss under photochemical reduction

The amount of Hg photoreductive loss (L, µg m^{-2} yr^{-1}) from surface peat was calculated as follows.

$$L_{sample} = Hg\,flux_{sample} * R_{sample} \qquad (6)$$

Where R_{sample}, based on the Rayleigh fractionation model (equation 7; Criss, 1999 [11]), represents the percentage of Hg loss relative to the total Hg flux in the sample.

$$R_{sample} = 1 - \exp\,(LN((1000 + a_{sample})/(1000 + b_{sample}))/(\alpha - 1)) \qquad (7)$$

Where a_{sample} stands for measured $\Delta^{199}Hg$ in the sample, and b_{sample} stands for the calculated Δ199Hg based on the $\Delta^{199}Hg$ vs $\Delta^{200}Hg$ between two atmospheric end members (i.e. atmospheric Hg^0 and rainfall Hg^{II}). α represents the isotopic fractionation factor, 1.00049 ($E^{199}Hg_{reactant/product}$ = 0.49, Yuan et al., 2019 [12]). The uncertainty of R_{sample} was estimated considering analytical uncertainties under Monte Carlo simulation.

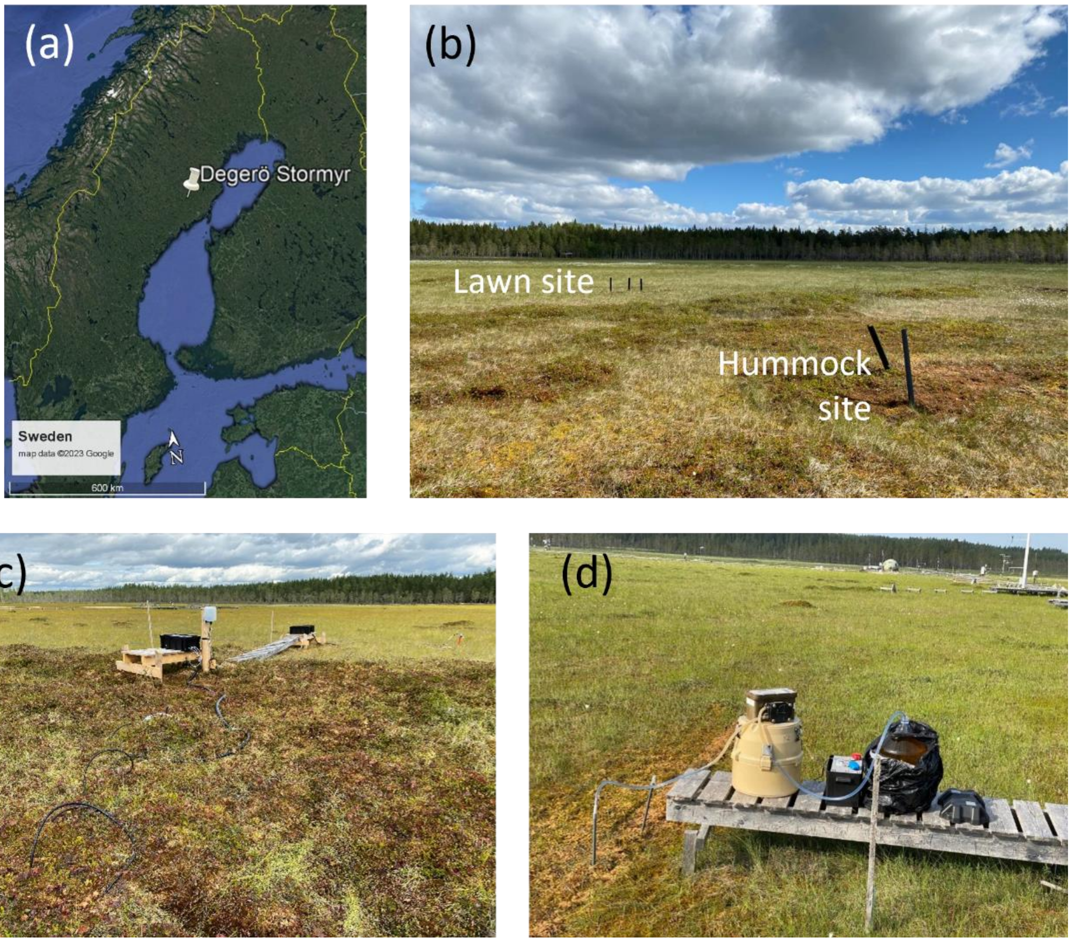

Figure S1. (a) Map of Sweden showing the location of Degerö Stormyr (white pin, map data ©2023 Google). (b) Locations of hummock and lawn sites for peat core collection (black sticks; hummock, 64°10'55.72"N, 19°33’33.21”E; lawn, 64°10’56.65”N, 19°33’32.52”E). (c) Site of peat gas and air sampling for Hg concentration and isotope analysis. (d) Site of peat groundwater sampling for dissolved gaseous elemental mercury (DGM) isotope analysis. Note that the glass bottle was fully wrapped in the black cover during peat groundwater sampling and transport. For photographing, the black cover was partially removed to reveal the glass bottle filled with peat groundwater. Photos b-d are taken by C.L.

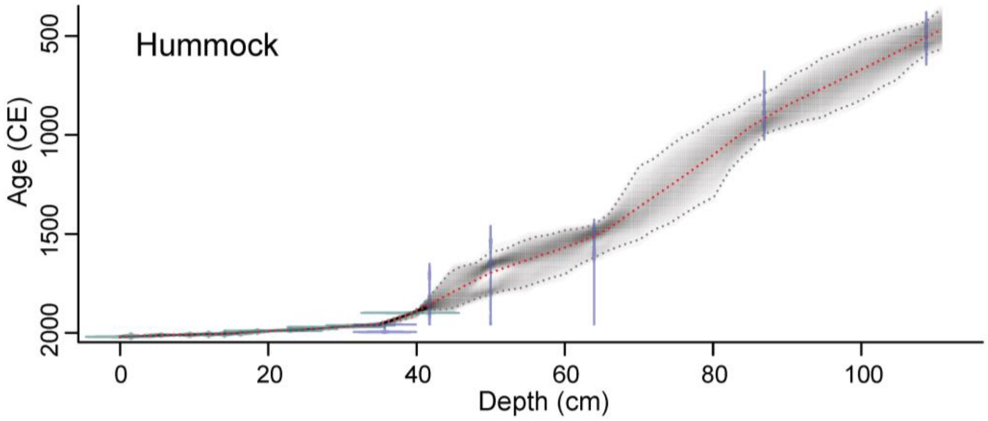

Figure S2. Calibrated age-depth model in hummock peat profiles under calibration curve IntCal20 [13]. The grey shaded area indicates 2SD confidence interval.

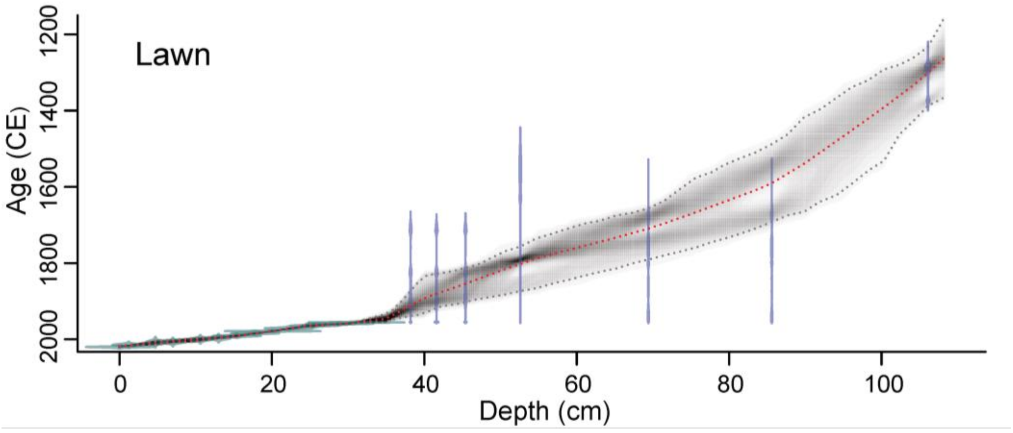

Figure S3. Calibrated age-depth model in lawn peat profiles under calibration curve IntCal20 [13]. The grey shaded area indicates 2SD confidence interval.

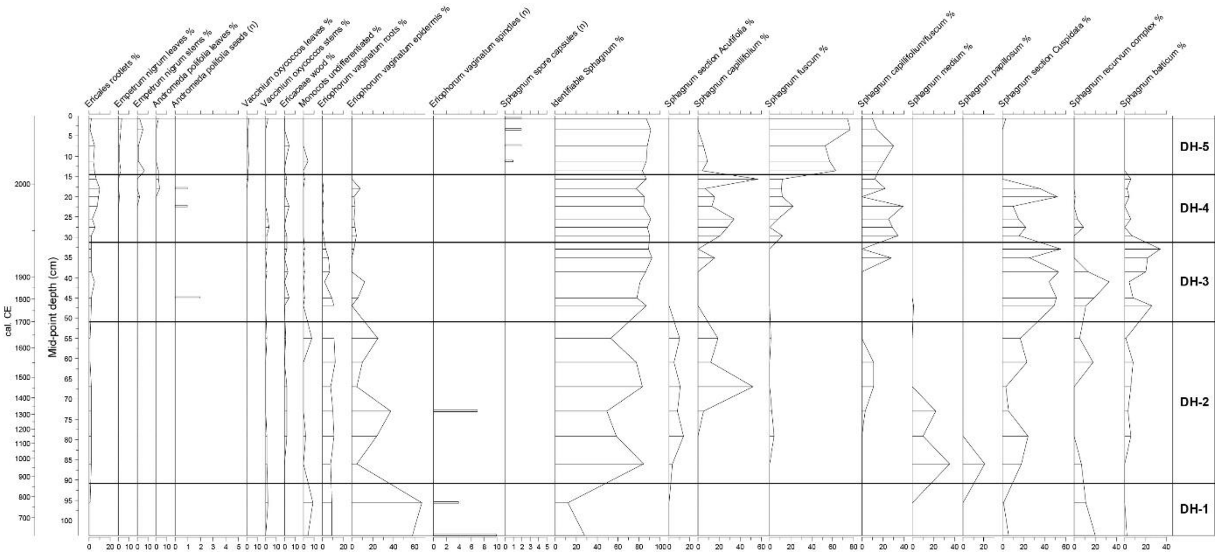

Figure S4. Plant macrofossil profile at the hummock site.

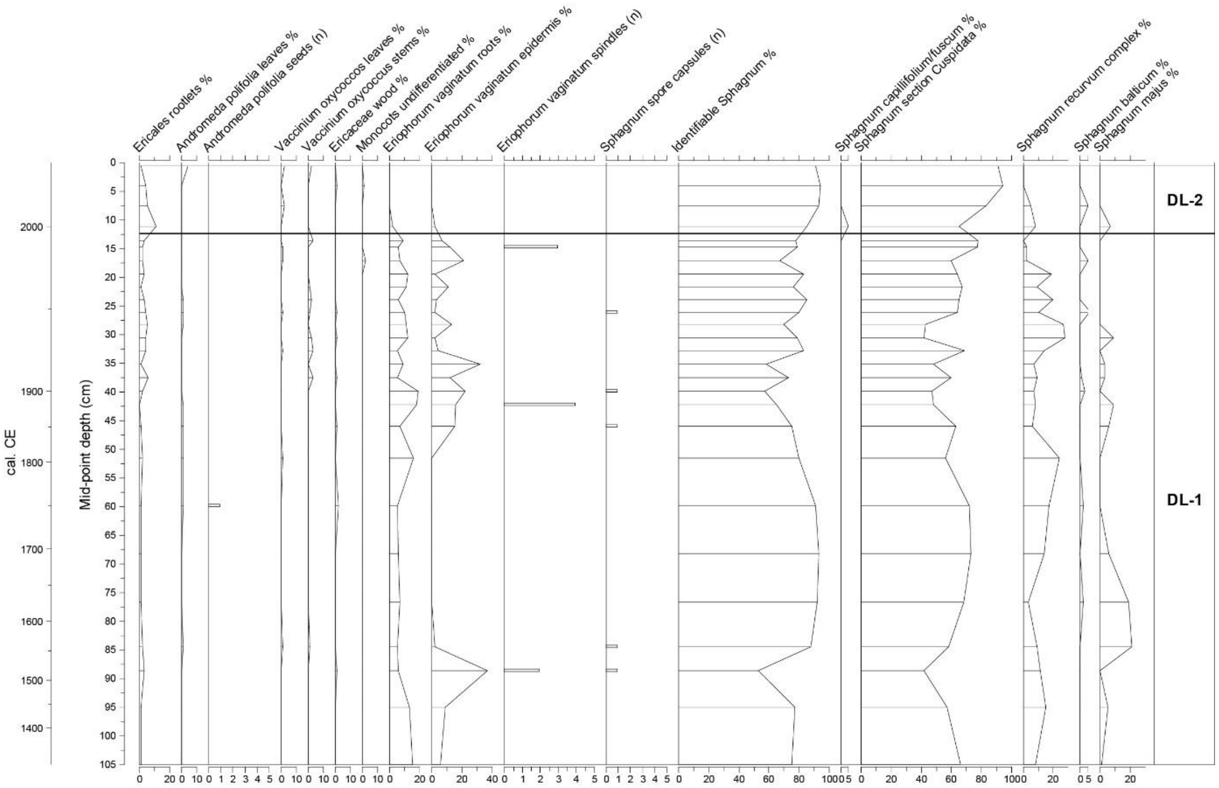

Figure S5. Plant macrofossil profile at the lawn site.

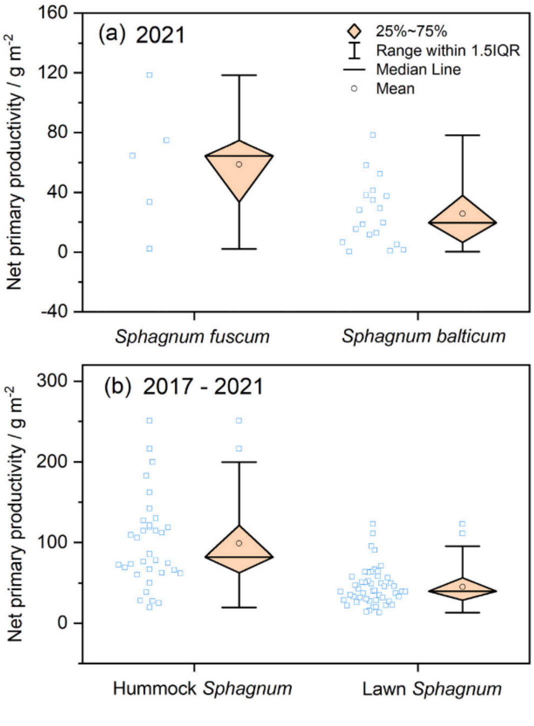

Figure S6. (a) The difference in net primary productivity (NPP) between *Sphagnum fuscum* and *Sphagnum balticum* in Degerö in 2021 ($P < 0.05$, two-tailed T test). (b) The difference in NPP between all the *Sphagnum* species in hummock and lawn from 2017 to 2021 considering NPP of each species and associated moss cover (S. papillosum, S. balticum, S. lindbergii, S. majus, S. fuscum, and S. medium). Data courtesy of ICOS Sweden.

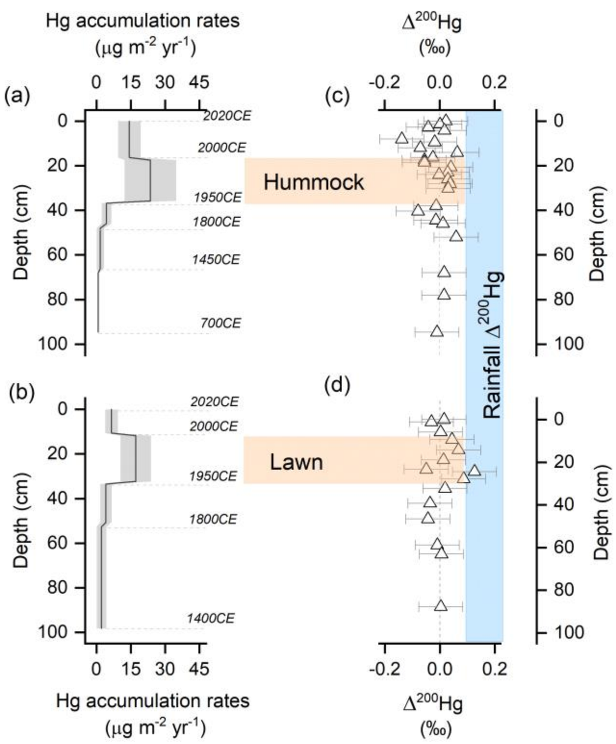

Figure S7. Profiles of calculated Hg accumulation rate (a, b, µg m^{-2} yr^{-1}) and MIF Δ^{200}Hg (c, d, ‰) in Degerö hummock peat (upper panel) and lawn peat (lower panel). Light orange shading in both upper and lower panels indicates the periods of 1950 − 2000 CE. The blue shading in Figure c and d represents the Northern Hemisphere remote areas rainfall Δ^{200}Hg signatures (0.16 ± 0.07‰, n = 55, refs please see the main text).

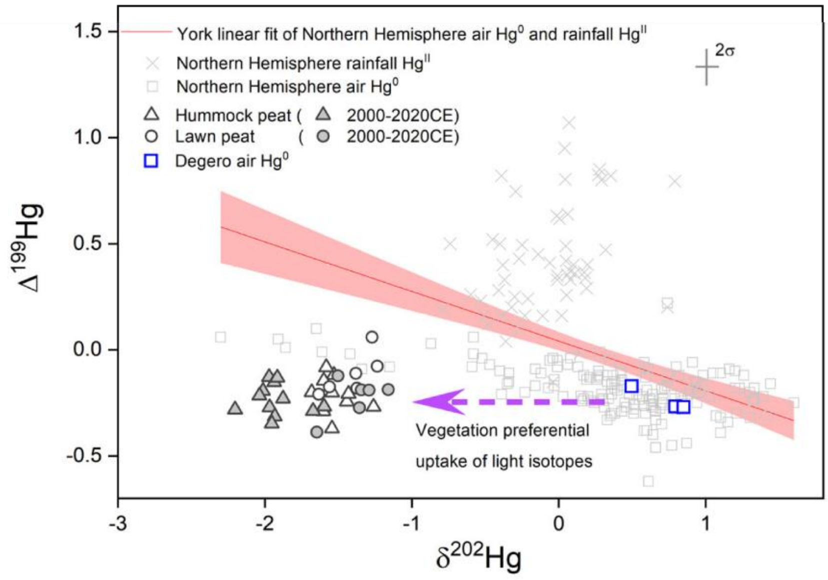

Figure S8. Δ^{199}Hg (‰) vs δ^{202}Hg (‰) in Northern Hemisphere rainfall Hg^{II} (light grey cross) and air Hg^0 (light open square), post− 1800CE hummock peat (black open triangle) and lawn peat (black open circle), and Degerö air Hg^0 (blue open square). Modern hummock and lawn peat (2000 − 2020CE) are highlighted in full symbols. The red line represents the York linear fit of Northern Hemisphere air Hg^0 and rainfall Hg^{II}. The light red shaded area stands for the 95% confidence band. The purple dashed arrow shows vegetation preferential uptake of light isotopes.

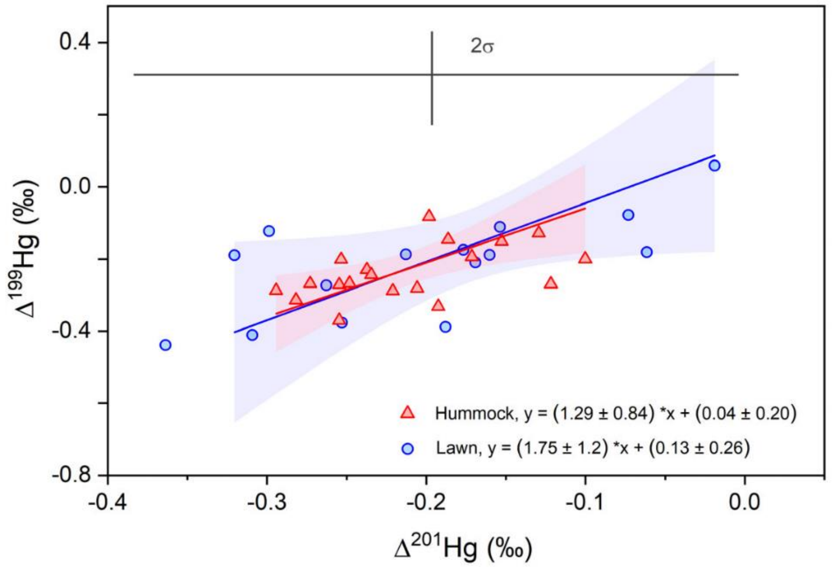

Figure S9. (b) Δ^{199}Hg (‰) vs Δ^{201}Hg (‰) in the top 1m profiles of both hummock (red triangles) and lawn (blue circles). Lines represent York linear fit of peat Δ^{199}Hg (‰) vs Δ^{201}Hg (‰) in hummock (red) and lawn (blue).

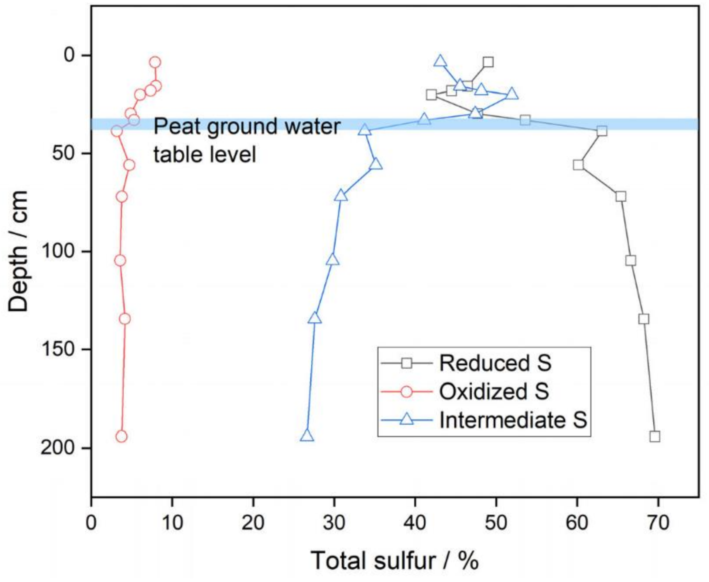

Figure S10. Depth profiles of intermediate sulfur (blue line), oxidized sulfur (orange line) and reduced sulfur (grey line) in the hummock profile. No sulfide is detected in the reduced group. The uncertainty is 5 - 10% [14].

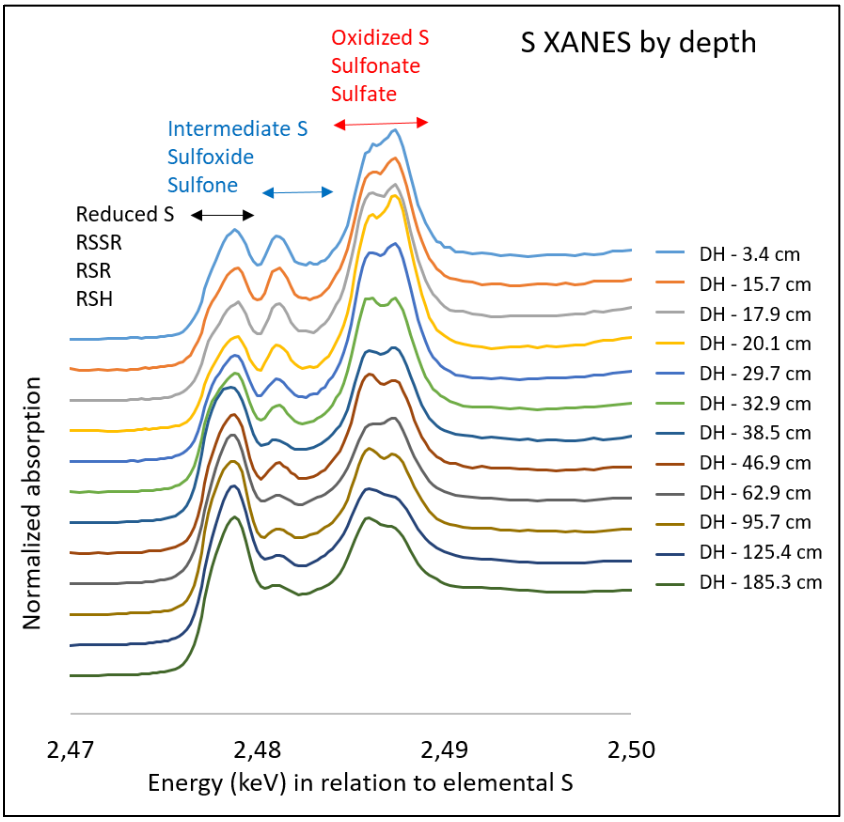

Figure S11. Normalized absorption of detailed Sulfur (S) species along depth profile for Reduced S (Organic sulfur (disulfide) / RSSR, sulfide / RSR and Organic sulfur (thiol) / RSH), Intermediate S (sulfoxide and sulfone) and Oxidized S (sulfonate and sulfate).

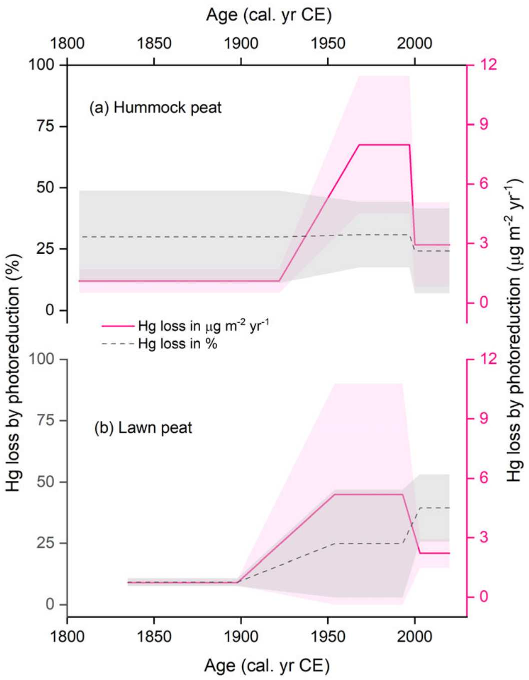

Figure S12. Temporal trends of photoreductive Hg loss in percentage (%, grey dash lines) and absolute amount (μg m^{-2} yr^{-1}, pink solid lines) for the peat profiles of hummock (a) and lawn (b). The shaded areas are the 1σ certainty bounds of these calculations for the losses in absolute (pink) and relative (grey) terms.

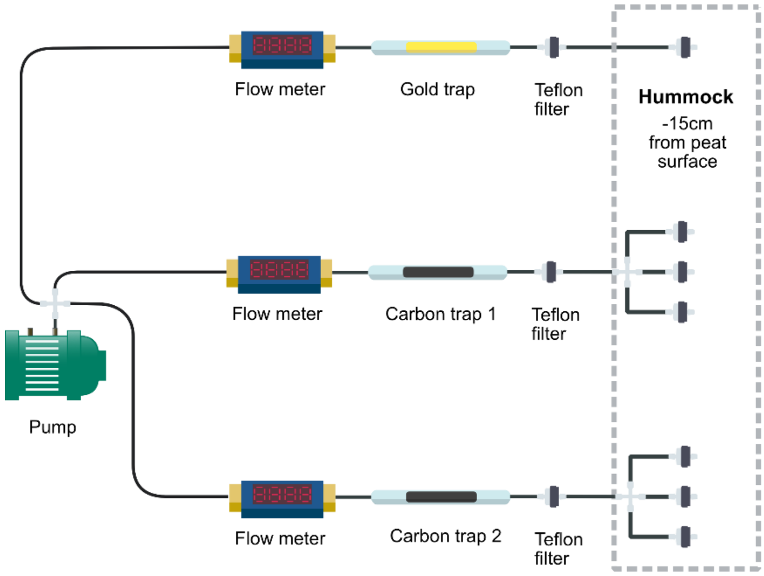

Figure S13. Schematic diagram of peat soil gas Hg^0 sampling in the Degerö hummock unsaturated zone (above water level). Sampling inlets were buried 15 cm below the peat surface. Upper line with one gold trap and one inlet was used to collect peat gas for Hg concentration analysis, while middle and lower lines with one carbon trap and three inlets each were used for Hg isotope measurements. A similar setup was designed for lawn peat gas and ambient air collection, with the intake at 10 cm depth below and 25 cm above the peat surface, respectively.

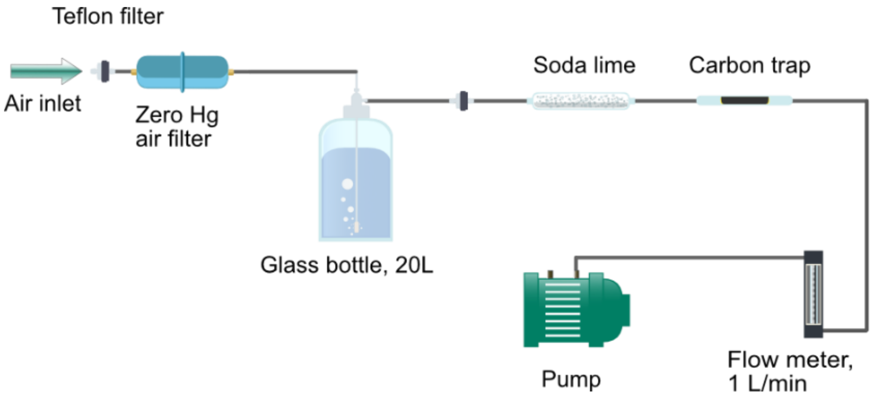

Figure S14. Schematic diagram of pre-concentration method for peat water dissolved gaseous elemental mercury (DGM) isotope analysis. Once collected and shipped back to the lab, peat water samples were started immediately for pre-concentration. A volume of 16 L peat water with 4 L headspace was used in a 20 L glass bottle for each pre-concentration.

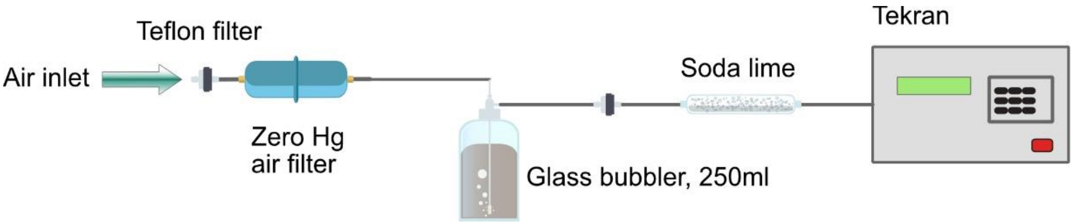

Figure S15. Schematic diagram of peat water and rainfall dissolved gaseous elemental mercury (DGM) concentration analysis using Tekran 2537X. A volume of 150 mL peat water was used in a 250 ml glass bubbler for each measurement.

Table S1. AMS ^{14}C dating of plant macrofossils from the top 1 m hummock (DH) and lawn (DL) peat cores from Degerö, Sweden

Lab code	Top depth (cm)	Material dated	^{14}C age (yr BP)	^{14}C content (pMC)	Calibrated age[1] (median, yr, CE)	Calibrated age[2] with ≥ 95.4% probablity (CE)
Ua-71010	DH-1.5	*Sphagnum capillifolium/fuscum* leaves,stems and branches	-137 ± 31	101.7 ± 0.4	2018	**2019 - 2014**; 1955
Ua-71011	DH-5.5	*Sphagnum capillifolium/fuscum* leaves,stems and branches	-299 ± 31	103.8 ± 0.4	2013	**2014 - 2009**; 1956 - 1955
Ua-71012	DH-9.4	*Sphagnum capillifolium/fuscum* leaves,stems and branches	-367 ± 31	104.7 ± 0.4	2010	**2013 - 2007**; 1957 - 1956
Ua-71013	DH-12	*Sphagnum capillifolium/fuscum* leaves,stems and branches	-437 ± 31	105.6 ± 0.4	2007	**2011 - 2004**; 1957 - 1956
Ua-71014	DH-14.1	*Sphagnum capillifolium/fuscum* leaves and stems	-490 ± 30	106.3 ± 0.4	2005	**2009 - 2003**; 1957 - 1956
Ua-71015	DH-16.3	*Sphagnum capillifolium/fuscum* leaves and stems	-622 ± 31	108.1 ± 0.4	2000	**2003 - 2000**; 1957
Ua-71016	DH-18.5	*Sphagnum fuscum* leaves and stems, *Empetrum nigrum* seeds	-985 ± 31	113.1 ± 0.4	1995	**1995 - 1991**; 1958
Ua-71017	DH-20.7	*Sphagnum fuscum* leaves and stems, *Empetrum nigrum* seeds	-1171 ± 30	115.7 ± 0.4	1990	**1990 - 1989**; 1959 - 1958
Ua-71018	DH-23	*Sphagnum capillifolium/fuscum* leaves and stems	-1402 ± 30	119.1 ± 0.5	1986	**1988 - 1985**; 1961 - 1958
Ua-71019	DH-26	*Sphagnum capillifolium/fuscum* leaves and stems	-1910 ± 30	126.9 ± 0.5	1980	**1981 - 1979**; 1959
Ua-71020	DH-29.2	*Sphagnum capillifolium*/section *Cuspidata* leaves and stems	-3357 ± 25	151.9 ± 0.5	1971	**1972 - 1969**; 1963
Ua-71021	DH-32.4	*Sphagnum capillifolium*/section *Cuspidata* leaves and stems	-2471 ± 25	136.0 ± 0.4	1964	**1962**; 1958 - 1957
Ua-71022	DH-35.7	*Sphagnum capillifolium/fuscum* leaves and stems	-899 ± 26	111.9 ± 0.4	1951	**1958 - 1957**; 1997 - 1993
Ua-71023	DH-39.1	*Sphagnum* section *Cuspidata* leaves and stems	-5 ± 28	100.1 ± 0.3	1907	**1907 - 1891**; 1720 - 1707; 1833 - 1815;1955 - 1953; 2017
Ua-71024	DH-41.8	*Sphagnum* section *Cuspidata* leaves and stems	116 ± 27	98.6 ± 0.3	1870	1908 - 1820
Ua-71025	DH-50	*Sphagnum* section *Cuspidata* leaves and stems	264 ± 26	96.8 ± 0.3	1742	1806 - 1622

Ua-71026	DH-64	*Sphagnum* section *Cuspidata* leaves and stems	361 ± 27	95.6 ± 0.3	1501	1624 - 1455
Ua-71027	DH-86.9	*Sphagnum papillosum* leaves and stems	1152 ± 28	86.6 ± 0.3	932	999 - 791
Ua-71028	DH-108.8	*Sphagnum* section *Cuspidata* leaves and stems	1562 ± 28	82.3 ± 0.3	493	583 - 423
Ua-71037	DL-1.2	*Sphagnum* section *Cuspidata* leaves,stems and branches	-186 ± 27	102.4 ± 0.3	2018	**2018 - 2012**; 1956 - 1955
Ua-71038	DL-4.7	*Sphagnum* section *Cuspidata* leaves,stems and branches	-427 ± 28	105.5 ± 0.4	2012	**2011 - 2005**; 1957 - 1956
Ua-71039	DL-7	*Sphagnum* section *Cuspidata* leaves,stems and branches	-458 ± 28	105.9 ± 0.4	2007	**2010 - 2003**; 1957 - 1956
Ua-71040	DL-10.6	*Sphagnum capillifolium/fuscum* leaves and stems	-705 ± 28	109.2 ± 0.4	2000	**2002 - 1997**; 1958 - 1957
Ua-71041	DL-13	*Sphagnum* section *Cuspidata* leaves and stems	-827 ± 27	110.9 ± 0.4	1996	**2000 - 1995**; 1958 - 1957
Ua-71042	DL-15.4	*Sphagnum* section *Cuspidata* leaves and stems	-1111 ± 27	114.8 ± 0.4	1991	**1993 - 1990**; 1958 - 1958
Ua-71043	DL-17.8	*Sphagnum* section *Cuspidata* leaves and stems	-1538 ± 27	121.1 ± 0.4	1985	**1985 - 1983**; 1962 - 1958
Ua-71044	DL-20.1	*Sphagnum* section *Cuspidata* leaves and stems	-2145 ± 26	130.6 ± 0.4	1979	**1979 - 1978**; 1962
Ua-71045	DL-22.2	*Sphagnum* section *Cuspidata* leaves and stems	-3177 ± 26	148.5 ± 0.5	1972	**1972 - 1970**; 1963 - 1962
Ua-71046	DL-25.6	*Sphagnum* section *Cuspidata* leaves and stems	-4700 ± 25	179.5 ± 0.6	1964	**1965 - 1963**
Ua-71047	DL-28.9	*Sphagnum* section *Cuspidata* leaves and stems, *Andromeda polifolia* leaves	-1737 ± 27	124.2 ± 0.4	1960	**1960 - 1959**; 1962 - 1961; 1983 - 7981
Ua-71048	DL-31.1	*Sphagnum* section *Cuspidata* leaves and stems	-214 ± 27	102.7 ± 0.3	1956	**1956 - 1955**; 2016 - 2012
Ua-71049[3]	DL-34.6	*Sphagnum* section *Cuspidata* leaves and stems	-461 ± 27	105.9 ± 0.4	1939	**1957 - 1956**; 2010 - 2003
Ua-71050	DL-38.2	*Sphagnum* section *Cuspidata* leaves and stems	78 ± 27	99.0 ± 0.3	1908	1936 - 1870
Ua-71051	DL-41.6	*Sphagnum* section *Cuspidata* leaves and stems	48 ± 27	99.4 ± 0.3	1879	1914 - 1832
Ua-71052	DL-45.4	*Sphagnum* section *Cuspidata* leaves and stems	55 ± 28	99.3 ± 0.3	1849	1896 - 1814

Ua-71053	DL-52.6	*Sphagnum* section *Cuspidata* leaves and stems	318 ± 27	96.1 ± 0.3	1793	1852 - 1762
Ua-71054	DL-69.4	*Sphagnum* section *Cuspidata* leaves and stems	173 ± 28	97.9 ± 0.3	1685	1753 - 1651
Ua-71055	DL-85.6	*Sphagnum* section *Cuspidata* leaves and stems	183 ± 28	97.8 ± 0.3	1546	1669 - 1481
Ua-71056	DL-106.1	*Sphagnum* section *Cuspidata* leaves and stems	706 ± 28	91.6 ± 0.3	1306	1387 - 1249

[1]Calibrated median age is derived from age-depth modeling performed with Bacon, Routine [13].
[2]Calibrated age range includes post-bomb calibrated age in hummock 0-39.1cm and lawn 0-36cm by Calibomb software of Queen's University , Belfast [15,16].
[3]Ua-71049 sample was deemed less reliable than the previous sample regarding ^{14}C age, so it is not used in modeling the ages. The median age and age range of this sample was derived from other dates using Bacon model.

Table S2. Profiles of depth, age, peat accumulation rate, density, Hg concentration and Hg isotope composition in both hummock and lawn sequences.

Core	Top depth (cm)	Calibrated median age (CE)	Peat accumulation rate (g cm^{-2} yr^{-1})	density (g cm^{-3})	c(Hg) (ng g^{-1})	$\delta^{204}Hg$ (‰)	$\delta^{202}Hg$ (‰)	$\delta^{201}Hg$ (‰)	$\delta^{200}Hg$ (‰)	$\delta^{199}Hg$ (‰)	$\delta^{198}Hg$ (‰)	$\Delta^{204}Hg$ (‰)	$\Delta^{201}Hg$ (‰)	$\Delta^{200}Hg$ (‰)	$\Delta^{199}Hg$ (‰)
Hummock	0.0	2020	0.032	0.041	35.7	-3.02	-1.97	-1.61	-0.97	-0.63	-3.24	-0.08	-0.13	0.02	-0.13
Hummock	1.5	2018	0.052	0.040	26.2	-2.92	-2.01	-1.69	-1.01	-0.70	-10.06	0.09	-0.17	0.00	-0.19
Hummock	2.8	2016	0.027	0.039	26.1	-2.95	-1.94	-1.61	-1.01	-0.64	-1.29	-0.06	-0.15	-0.04	-0.15
Hummock	4.2	2014	0.027	0.038	28.5	-2.79	-1.91	-1.95	-0.94	-0.61	1.89	0.07	-0.51*	0.02	-0.13
Hummock	5.5	2012	0.047	0.036	29.0										
Hummock	6.8	2011	0.049	0.037	34.4										
Hummock	8.1	2010	0.046	0.037	29.1	-3.23	-2.20	-1.33	-1.24	-0.84	-0.85	0.06	0.32*	-0.14	-0.28
Hummock	9.4	2009	0.027	0.038	29.5	-2.60	-1.97	-1.73	-1.01	-0.77	-5.00	0.34	-0.25	-0.02	-0.27
Hummock	10.7	2008	0.043	0.040	45.6										
Hummock	12.0	2007	0.042	0.043	44.1	-2.70	-1.93	-1.73	-1.04	-0.80	-1.03	0.19	-0.28	-0.07	-0.31
Hummock	13.0	2006	0.049	0.045	42.8										
Hummock	14.1	2005	0.024	0.051	47.1	-2.77	-1.88	-1.65	-0.88	-0.70	-3.16	0.03	-0.24	0.06	-0.23
Hummock	15.2	2003	0.016	0.061	49.5										
Hummock	16.3	2000	0.025	0.067	73.6	-2.27	-1.60	-1.45	-0.83	-0.67	1.14	0.12	-0.25	-0.02	-0.27
Hummock	17.5	1997	0.039	0.065	70.2	-2.39	-1.67	-1.55	-0.90	-0.71	-3.97	0.11	-0.29	-0.06	-0.29
Hummock	18.5	1995	0.022	0.062	64.1	-2.20	-1.44	-1.32	-0.78	-0.61	-1.89	-0.04	-0.23	-0.05	-0.24
Hummock	19.5	1992	0.034	0.061	57.8										
Hummock	20.7	1990	0.039	0.063	54.4	-2.55	-1.60	-1.33	-0.76	-0.67	-0.53	-0.16	-0.12	0.04	-0.27
Hummock	21.9	1988	0.033	0.065	49.0										
Hummock	23.0	1986	0.068	0.065	50.7	-2.41	-1.53	-1.01	-0.74	-0.50	-1.17	-0.12	0.14*	0.03	-0.12
Hummock	24.0	1984	0.038	0.068	62.0	-2.24	-1.43	-0.94	-0.72	-0.57	-5.67	-0.10	0.13*	0.00	-0.21
Hummock	25.1	1983	0.019	0.072	64.1										
Hummock	26.0	1980	0.025	0.077	73.0	-2.40	-1.68	-1.36	-0.81	-0.62	2.02	0.11	-0.10	0.03	-0.20
Hummock	27.0	1977	0.026	0.078	87.2										
Hummock	28.1	1974	0.028	0.074	98.3	-2.31	-1.60	-1.39	-0.76	-0.55	-2.51	0.08	-0.19	0.04	-0.15
Hummock	29.2	1971	0.028	0.073	106.9										
Hummock	30.2	1968	0.038	0.086	108.0	-2.30	-1.58	-1.39	-0.76	-0.48	-2.64	0.06	-0.20	0.03	-0.08

Hummock	31.4	1966	0.040	0.087	100.5										
Hummock	32.4	1964	0.064	0.108	71.4										
Hummock	33.5	1961	0.078	0.096	82.7										
Hummock	34.6	1959	0.015	0.105	74.7										
Hummock	35.7	1949	0.006	0.090	90.3										
Hummock	36.8	1934	0.008	0.105	84.6										
Hummock	38.0	1918	0.007	0.120	79.1	-2.33	-1.54	-1.41	-0.79	-0.59	1.01	-0.03	-0.25	-0.01	-0.20
Hummock	39.1	1901	0.009	0.127	82.4										
Hummock	40.4	1884	0.010	0.118	70.4	-2.45	-1.60	-1.43	-0.88	-0.69	1.90	-0.06	-0.22	-0.08	-0.29
Hummock	41.7	1860	0.008	0.112	70.8										
Hummock	43.1	1833	0.007	0.110	74.5										
Hummock	44.5	1803	0.008	0.110	49.7	-2.27	-1.54	-1.42	-0.79	-0.76	0.60	0.03	-0.25	-0.01	-0.37
Hummock	45.9	1767	0.006	0.112	34.4	-1.85	-1.26	-1.22	-0.62	-0.59	1.94	0.03	-0.27	0.01	-0.27
Hummock	47.9	1715	0.008	0.113											
Hummock	50.0	1662	0.007	0.121											
Hummock	52.0	1643	0.005	0.113	23.0	-1.64	-1.12	-1.05	-0.50	-0.56	-0.89	0.04	-0.21	0.06	-0.28
Hummock	54.0	1630	0.009	0.112	15.5										
Hummock	56.0	1606	0.008	0.110											
Hummock	58.1	1580	0.007	0.105											
Hummock	60.0	1556	0.007	0.101											
Hummock	61.9	1530	0.005	0.096	11.8										
Hummock	64.0	1501	0.005	0.095											
Hummock	66.0	1465	0.005	0.091	9.9										
Hummock	68.0	1421	0.004	0.102	19.0	-2.01	-1.41	-1.25	-0.69	-0.69	-7.96	0.10	-0.19	0.02	-0.33
Hummock	70.1	1375	0.003	0.116											
Hummock	72.1	1322	0.005	0.125	20.9										
Hummock	74.0	1271	0.005	0.117											
Hummock	76.1	1217	0.004	0.112											
Hummock	78.1	1152	0.004	0.108	16.7	-2.09	-1.41	-1.48	-0.69	-0.80	-0.29	0.02	-0.42	0.02	-0.45
Hummock	80.1	1088	0.005	0.108											
Hummock	82.2	1040	0.004	0.101											

Hummock	84.2	986	0.005	0.107											
Hummock	85.1	965	0.004	0.097	12.6										
Hummock	86.9	927	0.004	0.104											
Hummock	88.8	878	0.004	0.107											
Hummock	90.7	837	0.006	0.121											
Hummock	92.7	802	0.007	0.118											
Hummock	94.7	769	0.007	0.110	14.4	-2.05	-1.35	-1.46	-0.69	-0.81	-3.93	-0.03	-0.45	-0.01	-0.47
Hummock	96.7	732	0.006	0.104											
Hummock	98.7	691	0.006	0.103											
Lawn	0.0	2020	0.027	0.046	18.8	-2.51	-1.65	-1.43	-0.81	-0.80	-0.58	-0.06	-0.19	0.02	-0.39
Lawn	1.2	2016	0.023	0.038	21.7	-2.06	-1.36	-1.28	-0.71	-0.61	-2.72	-0.03	-0.26	-0.03	-0.27
Lawn	2.3	2013	0.028	0.044	22.4										
Lawn	3.6	2011	0.023	0.042	26.5										
Lawn	4.7	2009	0.025	0.044	22.7										
Lawn	5.8	2007	0.032	0.057	19.8	-1.87	-1.34	-1.17	-0.67	-0.53	-2.10	0.14	-0.16	0.00	-0.19
Lawn	7.0	2005	0.020	0.049	18.6										
Lawn	8.2	2004	0.029	0.050	23.4										
Lawn	9.4	2002	0.019	0.045	32.8	-1.65	-1.16	-1.09	-0.54	-0.48	-7.82	0.09	-0.21	0.04	-0.19
Lawn	10.6	2000	0.028	0.049	34.3										
Lawn	11.8	1998	0.029	0.046	49.1										
Lawn	13.0	1996	0.040	0.098	45.2										
Lawn	14.3	1993	0.028	0.048	67.2	-2.12	-1.29	-1.29	-0.58	-0.51	-9.15	-0.20	-0.32	0.07	-0.19
Lawn	15.4	1991	0.033	0.088	63.5										
Lawn	16.5	1988	0.024	0.058	59.1										
Lawn	17.8	1985	0.020	0.051	52.1										
Lawn	18.9	1982	0.035	0.093	61.2	-2.26	-1.50	-1.43	-0.74	-0.50	-7.94	-0.02	-0.30	0.01	-0.12
Lawn	20.1	1978	0.019	0.051	51.1										
Lawn	21.2	1975	0.013	0.051	46.1										
Lawn	22.2	1972	0.015	0.051	56.9										
Lawn	23.4	1968	0.021	0.061	73.8	-2.26	-1.37	-1.09	-0.74	-0.53	6.37	-0.21	-0.06	-0.05	-0.18
Lawn	24.5	1966	0.060	0.054	91.0	-1.94	-1.27	-0.97	-0.51	-0.26	-0.12	-0.05	-0.02	0.13	0.06

Lawn	25.6	1964	0.031	0.055	68.6										
Lawn	26.7	1962	0.051	0.047	55.2										
Lawn	27.8	1960	0.052	0.046	56.5	-1.93	-1.23	-1.00	-0.53	-0.39	-2.50	-0.09	-0.07	0.09	-0.08
Lawn	28.9	1959	0.023	0.040	63.1										
Lawn	30.0	1958	0.028	0.049	58.7										
Lawn	31.1	1956	0.027	0.046	71.2										
Lawn	32.3	1954	0.012	0.052	75.7	-2.07	-1.38	-1.19	-0.68	-0.46	-1.97	-0.01	-0.15	0.02	-0.11
Lawn	33.5	1949	0.008	0.060	63.6										
Lawn	34.6	1941	0.009	0.064	56.9										
Lawn	35.8	1933	0.007	0.064	65.3										
Lawn	37.0	1921	0.006	0.063	72.7										
Lawn	38.2	1909	0.009	0.070	72.3										
Lawn	39.3	1899	0.008	0.062	97.5	-2.30	-1.56	-1.35	-0.82	-0.57	2.38	0.03	-0.18	-0.04	-0.17
Lawn	40.4	1891	0.016	0.094	77.7										
Lawn	41.6	1883	0.008	0.070	78.8										
Lawn	42.8	1872	0.005	0.059	77.4										
Lawn	44.0	1859	0.007	0.068	77.1										
Lawn	45.4	1846	0.011	0.096	85.2										
Lawn	46.6	1836	0.009	0.087	88.9	-2.44	-1.63	-1.40	-0.86	-0.62	-1.84	0.00	-0.17	-0.04	-0.21
Lawn	47.4	1828	0.013	0.091											
Lawn	50.5	1805	0.014	0.094	73.4										
Lawn	52.6	1791	0.011	0.076	51.1										
Lawn	54.7	1778	0.012	0.076											
Lawn	56.8	1766	0.011	0.077											
Lawn	58.8	1752	0.010	0.064	27.8	-1.59	-1.09	-1.18	-0.56	-0.71	-2.76	0.03	-0.36	-0.01	-0.44
Lawn	61.0	1738	0.008	0.054	34.8										
Lawn	63.1	1723	0.014	0.091	47.7	-1.63	-1.20	-1.21	-0.60	-0.71	-3.68	0.16	-0.31	0.01	-0.41
Lawn	65.2	1709	0.010	0.064											
Lawn	67.3	1695	0.011	0.075	29.2										
Lawn	69.4	1681	0.010	0.069											
Lawn	71.5	1664	0.007	0.063											

Lawn	73.6	1647	0.007	0.056	11.1										
Lawn	75.7	1629	0.005	0.039											
Lawn	77.7	1612	0.006	0.052	13.4										
Lawn	79.3	1598	0.005	0.051											
Lawn	81.3	1580	0.006	0.050											
Lawn	83.5	1562	0.004	0.038	14.1										
Lawn	85.6	1541	0.007	0.072	14.7										
Lawn	87.7	1518	0.007	0.084	14.7	-2.39	-1.49	-1.37	-0.75	-0.75	-11.70	-0.16	-0.25	0.00	-0.38
Lawn	89.8	1496	0.006	0.070											
Lawn	91.9	1470	0.007	0.080											
Lawn	94.1	1445	0.007	0.070	6.3										
Lawn	96.1	1421	0.006	0.071											
Lawn	98.2	1393	0.005	0.065	10.0										

*$\Delta^{201}Hg$ in the hummock core with depths of 4.2, 8.1, 23 and 24cm are deemed as less reliable based on its generally significant relationship with $\Delta^{199}Hg$ under the same mechanisms (Blum et al., 2014)[17] and a better analytical performance of $\Delta^{199}Hg$ than $\Delta^{201}Hg$ in standard materials (supplementary table S7). We therefore do not include any $\Delta^{201}Hg$ data in the discussion.

Table S3. Hg concentration in different vegetation species from the Degerö peatland.

Item	Average (ng g^{-1})	std	mainly present in hummock/lawn
Scheuchzeria Palustris	3	0.3	lawn
Vaccinium oxycoccus	3	0.2	lawn
Andromeda polifolia	10	0.9	lawn
Eriophorum vaginatum	3	0.1	lawn
Sphagnum batticum	18	0.8	lawn
Sphagnum fuscum	25	0.5	hummock
Sphagnum lindbergii	31	2.2	Mainly in hummock, partially in lawn

Table S4. Atmospheric and peat gas Hg concentration at Degerö peatland over two summers in 2020 and 2021.

Position	name	Height (cm)	Start date	End date	Duration (h:min)	Inlet	Total volume (L)	flow of each inlet (lpm)	Hg concentration (ng m^{-3})
hummock	H1	-15			4:25	1	128	0.45	0.54
atmosphere	A1	25	8/14/2020	8/14/2020	4:28	1	123	0.45	1.52
lawn	L1	-10			4:28	1	123.6	0.45	0.67
hummock	H2	-15			5:42	1	155	0.45	0.49
atmosphere	A2	25	8/20/2020	8/20/2020	5:46	1	158.2	0.45	1.13
lawn	L2	-10			5:49	1	155.1	0.45	0.64
hummock	H3	-15			4:13	1	107.3	0.45	0.26
atmosphere	A3	25	8/26/2020	8/26/2020	4:13	1	113.7	0.45	1.29
lawn	L3	-10			4:12	1	110	0.45	0.29
hummock	H4	-15			5:40	1	151.1	0.45	0.24
atmosphere	A4	25	9/1/2020	9/1/2020	5:38	1	164.7	0.45	1.35
lawn	L4	-10			4:19	1	158.2	0.45	0.28
hummock	H5	-15			3:58	1	120.9	0.45	0.37
atmosphere	A5	25	9/4/2020	9/4/2020	4:00	1	126.4	0.45	1.38
lawn	L5	-10			3:57	1	123.3	0.45	0.46
hummock	H6	-15			4:03	1	132.9	0.45	0.32
atmosphere	A6	25	9/8/2020	9/8/2020	4:03	1	132.5	0.45	1.36
lawn	L6	-10			4:06	1	135.6	0.45	0.45
hummock	H7	-15			5:21	1	Failed. Water intrusion to the sampling tube		
atmosphere	A7	25	9/15/2020	9/15/2020	5:13	1	146.3	0.45	1.32
lawn	L7	-10			4:58	1	161.1	0.45	0.26
hummock	H8	-15			5:30	1	Failed. Leak during analysis		
atmosphere	A8	25	9/23/2020	9/23/2020	5:35	1	Failed. Glod trap broken		
lawn	L8	-10			5:36	1	148	0.45	0.59
hummock	H9	-15			6:53	1	198.2	0.45	0.26
atmosphere	A9	25	10/2/2020	10/2/2020	6:51	1	200.4	0.45	1.11
lawn	L9	-10			6:31	1	81.4	0.45	0.45

hummock	H10	-15			6:06	1	175.4	0.45	0.35
atmosphere	A10	25	10/8/2020	10/8/2020	6:05	1	175.2	0.45	1.30
lawn	L10	-10			6:05	1	Failed. Water intrusion to the sampling tube		
hummock	H11	-15	10/20/2020	10/20/2020	6:55	1	Failed. Water intrusion to the sampling tube		
atmosphere	A11	25			6:55	1	204.2	0.45	1.16
hummock	H12	-15			48:25	3	897.1	0.10	0.54
hummock	H13	-15			48:25	3	496.3	0.05	0.46
hummock	H14	-15	7/22/2021	7/24/2021	48:25	1	742.3	0.30	0.47
atmosphere	A12	25			48:25	1	950.9	0.30	1.38
lawn	L11	-10			48:25	3	583.6	0.07	0.52
hummock	H15	-15			9:25	3	137.0	0.10	0.46
hummock	H16	-15	7/24/2021	7/24/2021	9:25	3	82.2	0.05	0.38
hummock	H17	-15			9:25	1	141.5	0.30	0.17
atmosphere	A13	25			9:25	1	200.6	0.40	1.61
hummock	H18	-15			22:15	3	438.8	0.15	0.51
hummock	H19	-15			22:16	3	417.4	0.15	0.42
hummock	H20	-15	7/24/2021	7/25/2021	22:17	1	315.9	0.30	0.50
atmosphere	A14	25			22:18	1	139.0	0.15	1.63
lawn	L12	-10			22:19	3	417.5	0.15	0.53
hummock	H21	-15			15:46	3	176.0	0.05	0.59
hummock	H22	-15			15:47	3	418.0	0.15	0.52
hummock	H23	-15	7/25/2021	7/26/2021	15:48	1	444.5	0.45	0.58
atmosphere	A15	25			15:49	1	159.4	0.15	1.44
lawn	L13	-10			15:50	1	180.6	0.15	0.48
hummock	H24	-15			47:35	1	585.0	0.45	0.58
atmosphere	A16	25	7/26/2021	7/28/2021	47:36	1	569.7	0.45	0.97
lawn	L14	-10			47:37	1	369.4	0.45	0.59
hummock	H25	-15			30:20	1	330.0	0.45	0.55
atmosphere	3	25	7/28/2021	7/29/2021	30:20	1	356.5	0.45	1.25
lawn	L15	-10			30:20	1	345.3	0.45	0.52
hummock	H26	-15	8/4/2021	8/5/2021	24:10	1	253.2	0.45	0.34

atmosphere	A18	25			24:10	1	273.9	0.45	1.15
lawn	L16	-10			24:10	1	208.8	0.45	Failed in measurement
hummock	H27	-15			28:00	1	290.8	0.45	0.41
atmosphere	A19	25	8/16/2021	8/17/2021	28:00	1	228.6	0.45	1.25
lawn	L17	-10			28:00	1	Turned off due to high water table level		

Table S5. Sampling duration, volume of air, flow rate of each inlet, Hg concentration and Hg isotope composition in ambient air (noted as atmosphere) and peat soil gas samples (i.e. hummock and lawn).

Location	name	Height (cm)	Start date to end date	Inlet	time (d)	volume (L)	flow (lpm)	c(Hg) (ng m^{-3})	Hg amount (ng)	$\delta^{204}Hg$ (‰)	$\delta^{202}Hg$ (‰)	$\delta^{201}Hg$ (‰)	$\delta^{200}Hg$ (‰)	$\delta^{199}Hg$ (‰)	$\Delta^{204}Hg$ (‰)	$\Delta^{201}Hg$ (‰)	$\Delta^{200}Hg$ (‰)	$\Delta^{199}Hg$ (‰)
hummock	NO1_1	-15	8/11/2020 to 9/15/2020	3	35	25629	0.15	0.35	9.1	-0.39	-0.18	-0.18	-0.04	-0.12	-0.12	-0.04	0.05	-0.07
hummock	NO2_1	-15		3	35	27706	0.15	0.35	9.8	-0.78	-0.39	-0.85	-0.23	-0.08	-0.2	-0.56*	-0.04	0.02
lawn	NO6_1	25		3	35	23251	0.15	0.45	10.60	0.46	-0.03	-0.29	-0.31	-0.35	0.50	-0.27	-0.30	-0.35
atmosphere	NO4_1	-10		1	35	26703	0.45	1.29	32.69	1.44	0.85	0.44	0.36	-0.05	0.18	-0.2	-0.07	-0.27
hummock	NO1_2	-15	9/15/2020 to 10/20/2020	3	35	22030	0.15	0.31	6.72	-0.20	-0.15	-0.34	-0.15	-0.27	0.02	-0.23	-0.07	-0.23
hummock	NO2_2	-15		3	35	28832	0.15	0.31	8.79	0.15	0.02	-0.17	-0.04	-0.22	0.12	-0.18	-0.05	-0.23
lawn	NO6_2	25		3	35	Failed. Sampling tubing is waterlogged due to frequent rain and high water table level.												
atmosphere	NO4_2	-10		1	35	26797	0.45	1.22	34.60	1.37	0.79	0.29	0.23	-0.07	0.19	-0.31	-0.17	-0.27
hummock	NO1_3	-15	7/29/2021 to 9/15/2021	3	48	34778	0.15	0.33	11.41	0.33	0.19	-0.09	0.08	-0.11	0.05	-0.23	-0.02	-0.16
hummock	NO2_3	-15		3	48	36394	0.15	0.33	11.94	-0.16	-0.12	-0.15	-0.06	-0.07	0.02	-0.06	0.00	-0.04
lawn	NO6_3	25		3	48	Failed. Sampling tubing is waterlogged due to frequent rain and high water table level.												
atmosphere	NO4_3	-10		1	48	10993	0.15	1.07	11.66	0.94	0.50	0.23	0.18	-0.05	0.19	-0.15	-0.07	-0.17
hummock	NO1_4	-15	9/15/2021 to 10/20/2021	3	35	Failed. Sampling tubing is waterlogged due to frequent rain and high water table level.												
hummock	NO2_4	-15		3	35	Failed. Sampling tubing is waterlogged due to frequent rain and high water table level.												
atmosphere	NO4_4	-10		1	35	Failed in analysis.												

*$\Delta^{201}Hg$ in sample NO2_1 is deemed as less reliable based on its generally significant relationship with $\Delta^{199}Hg$ under the same mechanisms (Blum et al., 2014)[17] and a better analytical performance of $\Delta^{199}Hg$ than $\Delta^{201}Hg$ in standard materials (supplementary table S7). We therefore do not include any $\Delta^{201}Hg$ data in the discussion.

Table S6. Peat water volume, mean DGM concentration, Hg recovered from trap and sampling yield, and DGM isotope composition.

	Water volume L^{-1}	Mean $c(Hg^0)$* pg L^{-1}	Hg (ng)	Yield	$\delta^{204}Hg$ ‰	$\delta^{202}Hg$ ‰	$\delta^{201}Hg$ ‰	$\delta^{200}Hg$ ‰	$\delta^{199}Hg$ ‰	$\delta^{198}Hg$ ‰	$\Delta^{204}Hg$ ‰	$\Delta^{201}Hg$ ‰	$\Delta^{200}Hg$ ‰	$\Delta^{199}Hg$ ‰
DGM_1	480	24	11.5	80%	-0.92	-0.71	-1.67	-0.33	-0.70	7.75	0.15	-1.14	0.03	-0.52
DGM_2	482	24	11.6	92%	-1.03	-0.81	-1.01	-0.31	-0.66	-8.20	0.18	-0.40	0.10	-0.45
DGM_3	496	24	11.9	86%	-1.04	-0.73	-0.92	-0.31	-0.68	-5.60	0.05	-0.37	0.06	-0.49
DGM_4	480	24	11.5	84%	-1.18	-0.93	-1.08	-0.42	-0.70	-7.46	0.20	-0.38	0.04	-0.47

*Mean c(Hg0) was the average value of DGM concentration in the same peat water collected for isotope analysis at 30-50cm down from peat surface (n = 25). The “Hg recovered from the trap” is calculated by the measured DGM concentration and total volume of peat water for pre-concentration. The “sample yield” means the ratio of analyzed Hg concentration using CV-AFS method and Hg recovered from the trap.

Table S7. DGM concentration in rainfall and peat surface groundwater.

	DGM concentration / pg L^{-1}	Date of collection
Rainfall 1	20.45	7/5/2022
Rainfall 2	22.87	7/5/2022
Rainfall 3	26.05	7/5/2022
Rainfall 4	35.94	7/14/2022
Rainfall 5	31.16	7/14/2022
Rainfall 6	25.77	7/14/2022

Table S8. Hg isotopic composition of ETH-Fluka and Apple leaves.

Standard Type	Name	n	δ^{204}Hg (‰)	δ^{202}Hg (‰)	δ^{201}Hg (‰)	δ^{200}Hg (‰)	δ^{199}Hg (‰)	δ^{198}Hg (‰)	Δ^{204}Hg (‰)	Δ^{201}Hg (‰)	Δ^{200}Hg (‰)	Δ^{199}Hg (‰)	Refs
Secondary standard	ETH-Fluka	25	-2.16	-1.44	-1.07	-0.68	-0.29	-1.04	0.00	0.01	0.02	0.07	[18–22]
	2σ		0.28	0.12	0.22	0.35	0.13	6.16	0.23	0.16	0.08	0.10	
Procedural standard (Purge and Trap)	Apple Leaves (NIST SRM1515)	5	-4.07	-2.73	-2.11	-1.43	-0.65	0.59	0.00	-0.06	-0.06	0.03	[18,23]
	2σ		0.47	0.17	0.20	0.17	0.17	5.78	0.44	0.19	0.08	0.13	

Notes: *n* for ETH-Fluka is the number of analysis during two sessions from late Oct to early Dec 2021. *n* for Apple Leaves is the number of preparations of the materials for purging and trapping. *2σ* shows 2SD of the average of two sessions. The analytical performance on standards, except Δ^{201}Hg (higher uncertainty in our study), are well in line with published values shown in *Refs*.

Supplementary References

1. Clymo, R. S. The limits to peat bog growth. *Phil. Trans. R. Soc. Lond. B* **303**, 605–654 (1984).
2. Peichl, M. *et al.* Energy exchange and water budget partitioning in a boreal minerogenic mire: PEATLAND ENERGY AND WATER EXCHANGES. *J. Geophys. Res. Biogeosci.* **118**, 1–13 (2013).
3. Olid, C., Nilsson, M. B., Eriksson, T. & Klaminder, J. The effects of temperature and nitrogen and sulfur additions on carbon accumulation in a nutrient-poor boreal mire: Decadal effects assessed using ^{210}Pb peat chronologies. *J. Geophys. Res. Biogeosci.* **119**, 392–403 (2014).
4. Bengtsson, F., Granath, G. & Rydin, H. Photosynthesis, growth, and decay traits in *Sphagnum* – a multispecies comparison. *Ecol Evol* **6**, 3325–3341 (2016).
5. Millington, R. J. & Quirk, J. P. Permeability of porous solids. *Transactions of the Faraday Society* 1200–1207 (1961).
6. Päivänen, J. *Hydraulic conductivity and water retention in peat soils*. vol. 129 (Acta Forestalia Fennica, 1973).
7. Nijp, J. J. *et al.* Rain events decrease boreal peatland net CO_2 uptake through reduced light availability. *Glob Change Biol* **21**, 2309–2320 (2015).
8. Jones, H. G. *Plants and Microclimate: a Quantitative Approach to Environmental Plant Physiology*. (Cambridge University Press, New York, 1992).
9. Poissant, L., Amyot, M., Pilote, M. & Lean, D. Mercury Water−Air Exchange over the Upper St. Lawrence River and Lake Ontario. *Environ. Sci. Technol.* **34**, 3069–3078 (2000).
10. Andersson, M. E., Gårdfeldt, K., Wängberg, I. & Strömberg, D. Determination of Henry's law constant for elemental mercury. *Chemosphere* **73**, 587–592 (2008).
11. Criss, R. E. *Principles of Stable Isotope Distribution*. (Oxford University Press, 1999).
12. Yuan, W. *et al.* Stable Isotope Evidence Shows Re-emission of Elemental Mercury Vapor Occurring after Reductive Loss from Foliage. *Environ. Sci. Technol.* **53**, 651–660 (2019).
13. Blaauw, M. & Christen, J. A. Flexible paleoclimate age-depth models using an autoregressive gamma process. *Bayesian Anal.* **6**, (2011).
14. Song, Y. *et al.* Toward an Internally Consistent Model for Hg(II) Chemical Speciation Calculations in Bacterium–Natural Organic Matter–Low Molecular Mass Thiol Systems. *Environ. Sci. Technol.* **54**, 8094–8103 (2020).
15. Hua, Q. *et al.* ATMOSPHERIC RADIOCARBON FOR THE PERIOD 1950–2019. *Radiocarbon* **64**, 723–745 (2022).
16. Reimer, P. J. *et al.* The IntCal20 Northern Hemisphere Radiocarbon Age Calibration Curve (0–55 cal kBP). *Radiocarbon* **62**, 725–757 (2020).
17. Blum, J. D., Sherman, L. S. & Johnson, M. W. Mercury Isotopes in Earth and Environmental Sciences. *Annu. Rev. Earth Planet. Sci.* **42**, 249–269 (2014).
18. Demers, J. D., Blum, J. D. & Zak, D. R. Mercury isotopes in a forested ecosystem: Implications for air-surface exchange dynamics and the global mercury cycle: MERCURY ISOTOPES IN A FORESTED ECOSYSTEM. *Global Biogeochem. Cycles* **27**, 222–238 (2013).
19. Jiskra, M. *et al.* Mercury Deposition and Re-emission Pathways in Boreal Forest Soils Investigated with Hg Isotope Signatures. *Environ. Sci. Technol.* **49**, 7188–7196 (2015).
20. Obrist, D. *et al.* Tundra uptake of atmospheric elemental mercury drives Arctic mercury pollution. *Nature* **547**, 201–204 (2017).
21. Grigg, A. R. C., Kretzschmar, R., Gilli, R. S. & Wiederhold, J. G. Mercury isotope signatures of digests and sequential extracts from industrially contaminated soils and sediments. *Science of The Total Environment* **636**, 1344–1354 (2018).
22. Xu, H. M. *et al.* Mercury stable isotope compositions of Chinese urban fine particulates in winter haze days: Implications for Hg sources and transformations. *Chemical Geology* **504**, 267–275 (2019).
23. Scanlon, T. M. *et al.* Mercury Accumulation in Tree Rings: Observed Trends in Quantity and Isotopic Composition in Shenandoah National Park, Virginia. *J. Geophys. Res. Biogeosci.* **125**, (2020).
