# Supplementary material for: Mercury deposition and redox transformation processes in peatland constrained by mercury stable isotopes
Source: Nat Commun. 2023 Nov 15;14:7389. doi: 10.1038/s41467-023-43164-8 (PMC10652010; doi:10.1038/s41467-023-43164-8)
Supplement: Supplementary file 3 — Reporting Summary [file 41467_2023_43164_MOESM3_ESM.pdf]

nature portfolio

Corresponding author(s): Chuxian Li

Last updated by author(s): 2023-9-25

Reporting Summary

Nature Portfolio wishes to improve the reproducibility of the work that we publish. This form provides structure for consistency and transparency in reporting. For further information on Nature Portfolio policies, see our Editorial Policies and the Editorial Policy Checklist.

Statistics

For all statistical analyses, confirm that the following items are present in the figure legend, table legend, main text, or Methods section.

n/a	Confirmed	
☐	☒	The exact sample size (*n*) for each experimental group/condition, given as a discrete number and unit of measurement
☐	☒	A statement on whether measurements were taken from distinct samples or whether the same sample was measured repeatedly
☐	☒	The statistical test(s) used AND whether they are one- or two-sided<br>*Only common tests should be described solely by name; describe more complex techniques in the Methods section.*
☐	☒	A description of all covariates tested
☐	☒	A description of any assumptions or corrections, such as tests of normality and adjustment for multiple comparisons
☐	☒	A full description of the statistical parameters including central tendency (e.g. means) or other basic estimates (e.g. regression coefficient) AND variation (e.g. standard deviation) or associated estimates of uncertainty (e.g. confidence intervals)
☐	☒	For null hypothesis testing, the test statistic (e.g. *F*, *t*, *r*) with confidence intervals, effect sizes, degrees of freedom and *P* value noted<br>*Give P values as exact values whenever suitable.*
☐	☒	For Bayesian analysis, information on the choice of priors and Markov chain Monte Carlo settings
☐	☒	For hierarchical and complex designs, identification of the appropriate level for tests and full reporting of outcomes
☐	☒	Estimates of effect sizes (e.g. Cohen's *d*, Pearson's *r*), indicating how they were calculated

Our web collection on statistics for biologists contains articles on many of the points above.

Software and code

Policy information about availability of computer code

Data collection	No commercial, open source and custom code were used for data collection in this study.
Data analysis	No relevant software was used for data collection in this study.

For manuscripts utilizing custom algorithms or software that are central to the research but not yet described in published literature, software must be made available to editors and reviewers. We strongly encourage code deposition in a community repository (e.g. GitHub). See the Nature Portfolio guidelines for submitting code & software for further information.

Data

Policy information about availability of data

All manuscripts must include a data availability statement. This statement should provide the following information, where applicable:

- Accession codes, unique identifiers, or web links for publicly available datasets
- A description of any restrictions on data availability
- For clinical datasets or third party data, please ensure that the statement adheres to our policy

The data supporting the findings of this study are provided in the article and Supplementary Material.

Research involving human participants, their data, or biological material

Policy information about studies with human participants or human data. See also policy information about sex, gender (identity/presentation), and sexual orientation and race, ethnicity and racism.

Reporting on sex and gender	*Use the terms sex (biological attribute) and gender (shaped by social and cultural circumstances) carefully in order to avoid confusing both terms. Indicate if findings apply to only one sex or gender; describe whether sex and gender were considered in study design; whether sex and/or gender was determined based on self-reporting or assigned and methods used. Provide in the source data disaggregated sex and gender data, where this information has been collected, and if consent has been obtained for sharing of individual-level data; provide overall numbers in this Reporting Summary. Please state if this information has not been collected. Report sex- and gender-based analyses where performed, justify reasons for lack of sex- and gender-based analysis.*
Reporting on race, ethnicity, or other socially relevant groupings	*Please specify the socially constructed or socially relevant categorization variable(s) used in your manuscript and explain why they were used. Please note that such variables should not be used as proxies for other socially constructed/relevant variables (for example, race or ethnicity should not be used as a proxy for socioeconomic status). Provide clear definitions of the relevant terms used, how they were provided (by the participants/respondents, the researchers, or third parties), and the method(s) used to classify people into the different categories (e.g. self-report, census or administrative data, social media data, etc.) Please provide details about how you controlled for confounding variables in your analyses.*
Population characteristics	*Describe the covariate-relevant population characteristics of the human research participants (e.g. age, genotypic information, past and current diagnosis and treatment categories). If you filled out the behavioural & social sciences study design questions and have nothing to add here, write "See above."*
Recruitment	*Describe how participants were recruited. Outline any potential self-selection bias or other biases that may be present and how these are likely to impact results.*
Ethics oversight	*Identify the organization(s) that approved the study protocol.*

Note that full information on the approval of the study protocol must also be provided in the manuscript.

Field-specific reporting

Please select the one below that is the best fit for your research. If you are not sure, read the appropriate sections before making your selection.

☐ Life sciences ☐ Behavioural & social sciences ☒ Ecological, evolutionary & environmental sciences

For a reference copy of the document with all sections, see nature.com/documents/nr-reporting-summary-flat.pdf

Ecological, evolutionary & environmental sciences study design

All studies must disclose on these points even when the disclosure is negative.

Study description	In this study, we identify redox-related Hg post-depositional processes and associated magnitudes in an open (tree-less) boreal peatland system, Degerö Stormyr (64°11'N, 19°33'E). We have comprehensively investigated the uppermost meter of 14C dated peat soil by combining measurements of Hg concentrations with the natural abundance of Hg stable isotopes in key compartments of the peatland ecosystems (atmosphere, peat soil, groundwater and soil gas). These investigations are made in two distinct peat microforms, slightly elevated (20 – 30cm) hummocks and flatter lawns, which differ in the water table level relative to the peat surface and their characteristic vegetation composition. Both of these differences have the potential to affect Hg deposition rates and redox-related mobility processes. This study includes the very first reports of Hg concentration and isotopes in the peat soil gas of the unsaturated zone above the water table, as well as the Hg isotopes in dissolved Hg0 (DGM) of peat groundwater just below the water table.
Research sample	We have comprehensively investigated the uppermost meter of 14C dated peat soil by combining measurements of Hg concentrations with the natural abundance of Hg stable isotopes in key compartments of the peatland ecosystems (atmosphere, peat soil, groundwater and soil gas).
Sampling strategy	Based on the objectives of the study, established protocols (details please see method section), field situation and collectively >150 years' experience of all co-authors on peat study, we carefully carried out the sampling.
Data collection	We collected data following the established analytical procotols (details please see the Method section).
Timing and spatial scale	Data collection lasted from July 2020 to Dec 2021 in Degerö Stormyr (64°11'N, 19°33'E).
Data exclusions	Due to a large uncertainty measured in Δ201Hg data, we therefore do not include any Δ201Hg data in the discussion.
Reproducibility	All attempts to repeat the experiments were successfully when following associated protocols described in the Method section.

Randomization	It is not relevant to our study since we don't need to allocate data into groups.
Blinding	It is not relevant to our study since we don't need to use this method.

Did the study involve field work? ☒ Yes ☐ No

Field work, collection and transport

Field conditions	This study was conducted at the Degerö Stormyr, a Sphagnum-dominated minerotrophic open peatland, on areas without tree cover. The peatland covers two-thirds of the 6.5 km2 catchment area which is 270 m above sea level in the Kulbäcksliden Research Park (64° 11’N and 19°33’E). This is located in the municipality of Vindeln municipality, Västerbotten province, Sweden. The climate at Degerö peatland is cold temperate humid, with mean annual values of 523 mm for precipitation and +1.2°C for temperature (data from 1961-1990). Mean temperatures in July and January are +14.7°C and -12.4°C, respectively. The vegetation growing season is from May to October (156 ± 15 days based on 2001-2005). The rest of the year is characterized by snow cover, which in general reaches a depth of 0.6m. Previous work on Hg conducted in this peatland includes the influence of sulphate concentration on peat pore water Hg methylation, and Hg flux measurements. The Degerö peatland surface is dominated by lawns with minor occurrence of hummocks. More detailed information on the field conditions can be seen in Sect. 3.4 of the recently published work (https://www.frontiersin.org/articles/10.3389/feart.2023.1194749/full).
Location	Degerö Stormyr is 270 m above sea level in the Kulbäcksliden Research Park (64°11’N and 19°33’E).
Access & import/export	Our university has full access to this peatland to carry out sampling of peat, air and water.
Disturbance	Since this peatland is easy to access with previously established infrastructure and our field engineers are very supportive, we didn't meet any significant disturbance over the course of sampling.

Reporting for specific materials, systems and methods

We require information from authors about some types of materials, experimental systems and methods used in many studies. Here, indicate whether each material, system or method listed is relevant to your study. If you are not sure if a list item applies to your research, read the appropriate section before selecting a response.

Materials & experimental systems

n/a	Involved in the study	
☒	☐	Antibodies
☒	☐	Eukaryotic cell lines
☒	☐	Palaeontology and archaeology
☒	☐	Animals and other organisms
☒	☐	Clinical data
☒	☐	Dual use research of concern
☐	☒	Plants

Methods

n/a	Involved in the study	
☒	☐	ChIP-seq
☒	☐	Flow cytometry
☒	☐	MRI-based neuroimaging

Dual use research of concern

Policy information about [dual use research of concern]

Hazards

Could the accidental, deliberate or reckless misuse of agents or technologies generated in the work, or the application of information presented in the manuscript, pose a threat to:

No	Yes	
☒	☐	Public health
☒	☐	National security
☒	☐	Crops and/or livestock
☒	☐	Ecosystems
☒	☐	Any other significant area

Experiments of concern

Does the work involve any of these experiments of concern:

No	Yes	
☒	☐	Demonstrate how to render a vaccine ineffective
☒	☐	Confer resistance to therapeutically useful antibiotics or antiviral agents
☒	☐	Enhance the virulence of a pathogen or render a nonpathogen virulent
☒	☐	Increase transmissibility of a pathogen
☒	☐	Alter the host range of a pathogen
☒	☐	Enable evasion of diagnostic/detection modalities
☒	☐	Enable the weaponization of a biological agent or toxin
☒	☐	Any other potentially harmful combination of experiments and agents
